# 嵌合抗原受体T细胞免疫治疗T细胞恶性肿瘤研究进展

**DOI:** 10.3760/cma.j.issn.0253-2727.2022.04.015

**Published:** 2022-04

**Authors:** 护君 李, 开林 徐

**Affiliations:** 徐州医科大学血液病研究所，徐州医科大学附属医院血液科，江苏省骨髓干细胞重点实验室，徐州 221002 Blood Diseases Institute, Xuzhou Medical University; Department of Hematology, the Affiliated Hospital of Xuzhou Medical University; Key Laboratory of Bone Marrow Stem Cell, Xuzhou 221002, China

复发难治T细胞恶性肿瘤目前尚无特效药物，临床前研究及临床研究证实嵌合抗原受体T细胞（CAR-T细胞）治疗可能成为复发难治T细胞恶性肿瘤最有前景的免疫治疗手段之一，但靶向T细胞肿瘤的CAR-T尚面临巨大挑战：①无特异性靶抗原：理想状态下，靶抗原应仅表达于肿瘤细胞上，而在正常T细胞上不表达，对于T细胞恶性肿瘤来说，大多数靶抗原在正常T细胞和肿瘤T细胞均表达；②难以区分正常T细胞和肿瘤T细胞：CAR-T细胞疗法需要从患者体内分离出正常T细胞，否则很可能导致产品污染或肿瘤细胞的CAR修饰[1]；③CAR-T细胞自相残杀：靶抗原在CAR-T细胞上的表达会导致CAR-T细胞自相残杀，而靶向正常T细胞表达的抗原会引起T细胞免疫缺陷，进而导致严重的机会性感染[2]。本文对CAR-T细胞治疗T细胞恶性肿瘤的临床前及临床研究综述如下。

一、靶抗原及相应CAR-T细胞的有效性

1. CD5：CD5在80％的未成熟的急性T淋巴细胞白血病（T-ALL）和成熟的T细胞淋巴瘤（TCL）中表达，在正常胸腺细胞、正常T细胞和一小部分B淋巴细胞也有所表达，正常造血细胞不表达[3]–[4]。既往研究也证实了CD5抗体治疗皮肤T细胞淋巴瘤和T-ALL的安全性和有效性[5]。

研究者发现构建含有CD28共刺激结构域的二代靶向CD5的CAR导致CAR-T细胞CD5表达丧失，因此CD5 CAR-T细胞无明显自相残杀。体外实验表明靶向CD5 CAR-T细胞对CD5^+^ T细胞系和肿瘤细胞具有强大的细胞毒性，而对正常活化的CD5^+^ T细胞毒性有限，可能是由于正常T细胞对其自身的细胞毒性机制具有较高的固有抵抗力[6]–[7]。

MAGENTA研究[7]共纳入9例患者，包括4例CD5^+^复发难治T-ALL和5例TCL患者，总有效率（ORR）为44％（4/9），其中3例获得完全缓解（CR）。无严重细胞因子释放综合征（CRS），其中2例患者出现长期全血细胞减少，1例患者巨细胞病毒（CMV）和BK病毒再激活，需要抗病毒治疗。

上述研究的初步结果表明CD5 CAR-T细胞是安全的，可以诱导对复发难治CD5^+^ T-ALL和TCL患者的临床反应，并且不会诱导完全性T细胞免疫抑制。该研究病例数少，随访时间短，需要更多的研究进一步证实。

2. CD7：CD7表达在95％的T-ALL和TCL上[3]，也表达在大多数外周T细胞和NK细胞上。在小鼠模型中，缺乏CD7的T细胞仍能维持正常发育、体内平衡和保护功能[8]，并且目前研究并未阐明CD7对外周T细胞的关键功能，因此CD7可能是CAR-T的一个有潜力的靶标。然而，因大多数正常T细胞均表达CD7，CAR修饰的T细胞会出现致命的自相残杀导致体内外无法扩增。

研究者通过编辑敲除CD7基因或阻断CD7蛋白运输到细胞表面阻止CD7表达，而消除CD7表达并未影响T细胞增殖和T细胞的短期效应子功能，仍然保留抗肿瘤活性[9]–[10]。因此，在体内外试验中靶向CD7的CD7^−^ CAR-T 细胞可迅速扩增，且对CD7^+^ T细胞肿瘤发挥强大的抗肿瘤活性[11]–[12]。研究者也观察到CAR-T细胞对外周 T细胞和NK细胞的杀伤作用，但输注的抗CD7 CAR-T细胞可能通过其天然受体保留抗病毒活性，从而抵消抗CD7 CAR-T 细胞脱靶诱导的严重免疫缺陷[10]。目前评估自体或异基因靶向CD7 CAR-T细胞的临床试验在国内外开展，且初步结果也显现出抗CD7 CAR-T细胞在T细胞恶性肿瘤中良好的应用前景。

在2020年欧洲血液学协会（EHA）年会上郑州大学第一附属医院报告了5例患者［4例为T-ALL/淋巴母细胞淋巴瘤（LBL），1例为NK/T细胞淋巴瘤］接受抗CD7 CAR-T细胞治疗后，4例获得了CR，1例患者获得了部分缓解（PR），其中1例在获得CR后死于腹腔感染。1例患者出现谷氨酸氨基转移酶升高，最高达990 U/L，28 d后逐步恢复正常[11]。

高博团队研究报道了供者来源CD7 CAR-T细胞治疗T-ALL的研究结果：入组20例患者，ORR达95％（19/20），微小残留病（MRD）阴性的CR率达85％。中位随访6.3（4～9.2）个月，19例获得临床缓解的患者中，7例接受了造血干细胞移植，均重建造血系统，其中6例仍保持无疾病生存（DFS）状态；11例未桥接治疗的CR患者中，9例患者仍处于DFS状态，1例在5.5个月时死亡，1例在4个月时CD7阴性复发。研究显示异基因CD7-CAR-T联合异基因造血干细胞移植（allo-HSCT）有良好的疗效和安全性[12]。

2021年美国癌症研究协会（AACR）年会以海报形式公布了一项基于TruUCAR平台开发的通用型同种异基因候选产品GC027治疗复发难治T-ALL成人患者的最新长期随访数据[13]。结果显示，入组的6例患者均达到CR，其中5例MRD阴性。1例患者治疗前伴有高肿瘤负荷及广泛髓外病变，在接受了TruUCAR GC027治疗后，所有髓外病灶消失，并在28 d达到MRD阴性。所有患者都出现了不同程度的可控制的CRS，未观察到神经毒性事件（ICANS）或急性移植物抗宿主病（aGVHD）的发生。

3. CD3：CD3在大多数TCL和一部分T-ALL细胞表面表达，在正常组织中，其表达限于T细胞和胸腺细胞[14]。CD3单克隆抗体已在T细胞淋巴瘤的治疗中应用，其耐受性良好但疗效持续时间短[15]。与CD7一样，靶向CD3的CAR-T会导致自我靶向而自相残杀，因此也需要消除CD3的表达。靶向CD3的抗CD3^−^CAR-T 细胞在体内外迅速扩增并清除CD3^+^ T细胞肿瘤。然而，抗CD3 CAR-T细胞可能会促进正常T细胞上的T细胞抗原受体（TCR）交联，可导致T细胞活化而排斥CAR-T细胞[16]。此外CD3也表达在正常T细胞上，治疗后可能出现T细胞免疫缺陷。因此其安全性和有效性仍需要在临床研究中进行评估。

4. CD4：CD4在大多数TCL以及一部分T-ALL表达，仅在一部分成熟T细胞上表达，使其成为免疫治疗的另一个潜在靶点。CD4单克隆抗体已在临床上应用于多种自身免疫性疾病以及皮肤和外周T细胞淋巴瘤，具有良好的耐受性和可逆性，无明显免疫抑制。鉴于此，研究者构建靶向CD4的CAR-T细胞，结果导致CD4^+^ T细胞自相残杀和抗CD4 CD8^+^ CAR-T 细胞的富集[17]。实验表明抗CD4 CD8^+^ CAR-T细胞对CD4肿瘤细胞系具有良好的细胞毒性。2016年8月，美国FDA已经授予iCell Gene Therapeutics公司在研抗CD4 CAR-T为治疗外周T细胞淋巴瘤（PTCL）的孤儿药资格。然而这种靶向CD4的CAR-T细胞会诱导CD4^+^ T细胞发育不全，从而导致艾滋病样综合征。目前抗CD4 CAR-T细胞治疗其他T细胞恶性肿瘤的临床试验正在开展和评估中。

5. CD1a：CD1a表达在35％～40％的皮质T-ALL细胞，也表达在发育中的皮质胸腺细胞、皮肤朗格汉斯细胞和一些循环髓样树突状细胞，正常T细胞和CD34^+^造血祖细胞均不表达CD1a[18]–[19]。研究表明，构建的抗CD1a CAR-T细胞没有自相残杀，可以在体内持续存在。此外，抗CD1a CAR-T细胞在体外表现出对CD1a^+^ T-ALL细胞系和原代原始细胞的特异性细胞毒活性，在皮质T-ALL的人源肿瘤异基因移植模型中也表现出有效的抗白血病活性。因此，抗CD1a CAR-T细胞在CD1a^+^ T细胞肿瘤中可能会取得良好的效果。

6. CD30：CD30表达在霍奇金淋巴瘤（HL）、间变性大细胞淋巴瘤（ALCL）和约1/3的T-ALL上[20]。Ramos等[21]报道了9例复发难治CD30淋巴瘤患者的结果（7例HL，1例为皮肤ALK^−^ ALCL，1例为全身性ALK^+^ ALCL）。ORR为33％（3/9），其中ALK^+^ ALCL患者获得CR，ALK^−^ ALCL无效。所有患者在治疗过程中出现可控制的CRS，未发现ICANS。目前国内外临床试验正在评估抗CD30 CAR-T细胞治疗T细胞肿瘤的安全性和有效性。

7. CD37：CD37是一种跨膜蛋白，血管免疫母细胞性T细胞淋巴瘤、ALCL、T-ALL、PTCL和所有结外、鼻型NK/T细胞淋巴瘤均有该靶点表达，正常表达主要局限在成熟B细胞上，浆细胞和树突细胞中表达较低[22]。CD37单克隆抗体药物偶联物的Ⅰ期临床试验证实其在一些皮肤T细胞淋巴瘤和PTCL患者中显示出良好的耐受性和有效性[23]。抗CD37 CAR-T细胞在治疗CD37^+^ T细胞恶性肿瘤的临床前研究模型中表现出有限的自相残杀效应和有效的抗肿瘤活性[24]。

8. TRBC1：正常T细胞可表达编码TCR β链恒定区的两个基因之一（TRBC1或TRBC2）。约50％的 TCR^+^ T细胞淋巴瘤仅表达TRBC1，靶向TRBC1的CAR-T细胞可以特异性地消除TRBC1^+^肿瘤细胞，而保留表达TRBC2的正常T细胞。因此，靶向TRBC1可能有益于TRBC1^+^ T细胞恶性肿瘤患者。然而，与靶向CD3的CAR-T细胞一样，抗TRBC1 CAR-T细胞对T细胞TCR的潜在交联可能会降低CAR-T细胞的持久性和抗肿瘤活性[25]。其安全性和有效性仍需进一步临床试验来评价。

9. CCR4：CCR4表达在成人T-ALL以及大多数皮肤T细胞淋巴瘤（PTCL、皮肤T细胞淋巴瘤、真菌病/Sezary综合征）和ALCL，但正常表达主要局限在调节性T细胞（Treg）、Th2和Th17细胞以及血小板上。研究表明CCR4 CAR-T细胞在体内外对一些高水平表达CCR4的恶性T细胞系显示出明显的抗肿瘤活性[26]。然而，在接受CCR4单克隆抗体治疗的患者中已有报告Treg耗竭相关的严重皮肤毒性病例。因此，抗CCR4 CAR-T的安全性需进一步的研究评估。

二、如何区分正常T细胞和肿瘤T细胞

针对自体CAR-T，利用T-ALL/LBL肿瘤细胞一般不表达CD4、CD8的特性，郑州大学第一附属医院提取CD4^+^或CD8^+^正常T细胞，避免了产品污染和肿瘤T细胞被CAR修饰[11]。对于CD4^+^ T细胞恶性肿瘤，则需要其他方法分离正常T细胞，有研究者通过纯化非T细胞制备CAR-T细胞，例如NK细胞[27]。

异基因CAR也是一个很好的选择。应用异基因供者来源T细胞制备CAR-T需进行供受者HLA配型，警惕致命性GVHD的发生，最近Pan等[12]应用HLA相合供者来源T细胞制备的抗CD7 CAR-T细胞在临床试验中显示出良好的疗效。通过基因编辑敲除TCR可以有效控制GVHD的发生，使通用型CAR-T的开发应用变为可能，例如通用型同种异基因候选产品GC027[13]。

三、如何避免CAR-T细胞的自相残杀和T细胞免疫缺陷

1. 基因编辑敲除目标基因：通过使用基因编辑（如转录激活样效应因子核酸酶或规律成簇的间隔短回文重复系统[9]）敲除目标基因来避免自相残杀，这种方法在CD7 CAR-T细胞中进行了临床前评估。也可应用CRISPR/Cas9介导的CD7编辑废除自相残杀效应并使CAR-T细胞扩增。研究者使用 PEBL技术也获得了类似的结果，该技术通过将新合成的CD7锚定在内质网和（或）高尔基体来阻止其在细胞表面表达[28]。2020 EHA年会有研究报道通过靶向CD7的纳米抗体识别糖基化的CD7抗原，融合PEBL序列诱导CD7停留在胞内，有效避免了CAR-T的自相残杀，且保留了较强的抗肿瘤效应[29]。

2. 构建CAR-NK细胞：NK细胞以CD56和CD7表达为特征，缺乏TCR、CD3和CD5表达，因此使用NK细胞是避免自相残杀的一个有前途的策略[30]。然而，NK细胞的收集、扩增和转导存在困难。鉴于NK-92细胞系已被批准应用到临床，抗CD3、CD4和CD5的CAR-NK细胞已经采用NK-92 细胞系替代NK细胞[27],[31]–[33]。在临床前研究中，CAR NK-92细胞可以显著降低肿瘤负荷，但在异种移植小鼠模型中缺乏持久性。临床前研究显示小鼠在输注抗CD3和CD5 CAR-NK-92细胞后出现ICANS[34]。此外，由于 CAR NK-92细胞起源于转化细胞系，可能存在潜在致瘤性[32]–[33]。因此，仍需要进一步研究以优化CAR-NK细胞疗法。

3. 避免T细胞免疫缺陷：可能有效的应对方案如下：①选用在正常T细胞上不表达的抗原，如CD1a、CD37或其他新的特异性抗原，但临床应用存在局限性；②CAR-T细胞制备前提取CD7^−^ T淋巴细胞扩增，待出现T细胞免疫缺陷后回输CD7^−^ T细胞[11]；③桥接allo-HSCT；④使用短寿命CAR-T细胞，例如带有“自杀基因”的CAR-T细胞[35]–[36]，但可能影响CAR-T细胞的疗效和持续时间。

四、小结

T细胞肿瘤的CAR-T细胞疗法面临多种挑战，由于存在自相残杀现象，需要通过有效手段降低CAR-T细胞靶抗原的表达或构建非T细胞的CAR。此外，自体CAR-T的构建可能出现CAR-T产品污染，未来研究可能会向异基因CAR-T的治疗方向发展。虽然靶向CD5、CD30及CD7等靶抗原的CAR-T在T细胞肿瘤中已取得初步的疗效，但其安全性仍需进一步评估。
